# Supplementary figures and images for: Executive functions mediate the relationship between cardiorespiratory fitness and academic achievement in Spanish schoolchildren aged 8 to 11 years
Source: PLoS One. 2020 Apr 10;15(4):e0231246. doi: 10.1371/journal.pone.0231246 (PMC7147757; doi:10.1371/journal.pone.0231246)

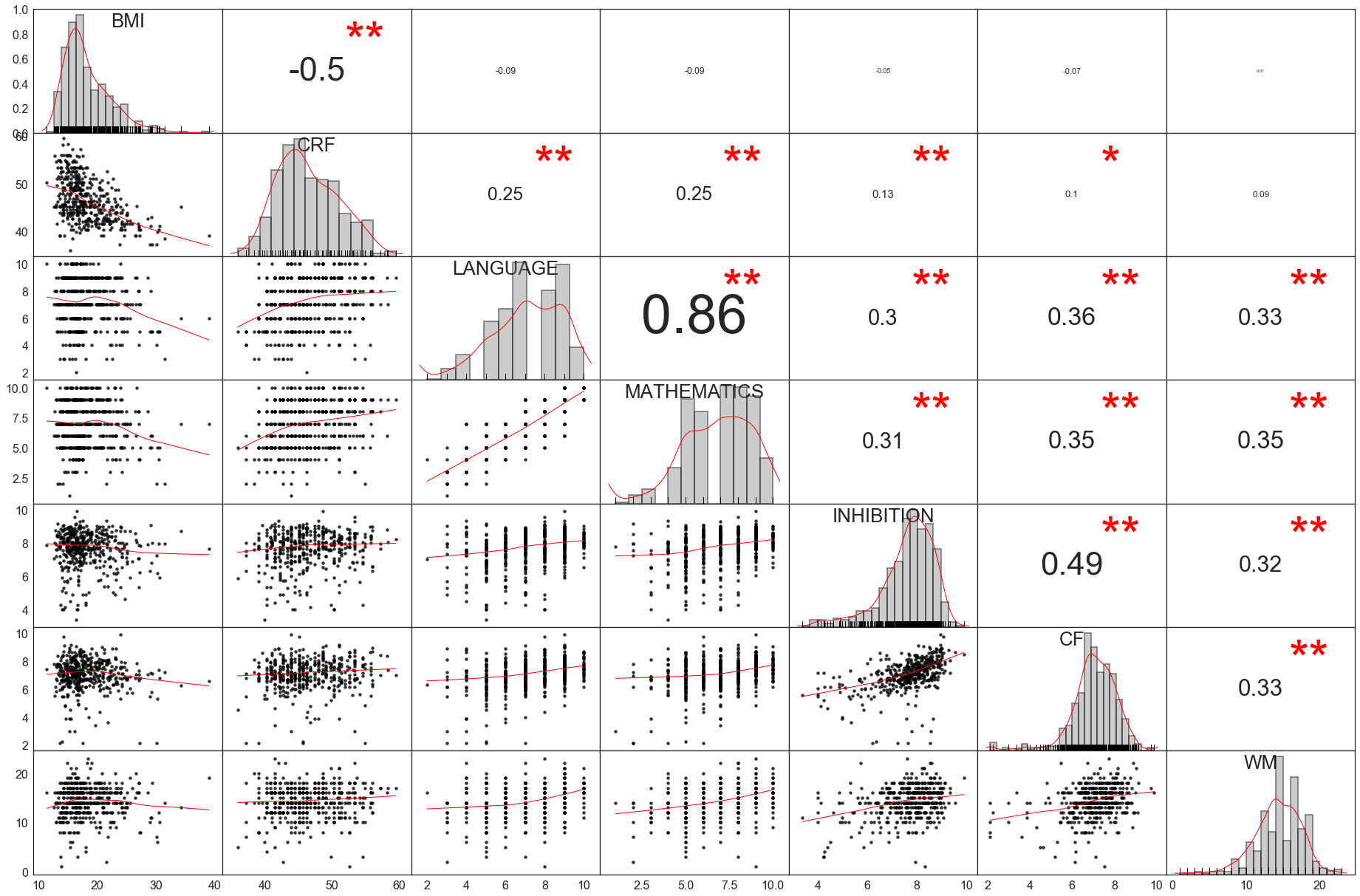

Supplement: S1 Fig — (TIFF) [file pone.0231246.s001.tiff]

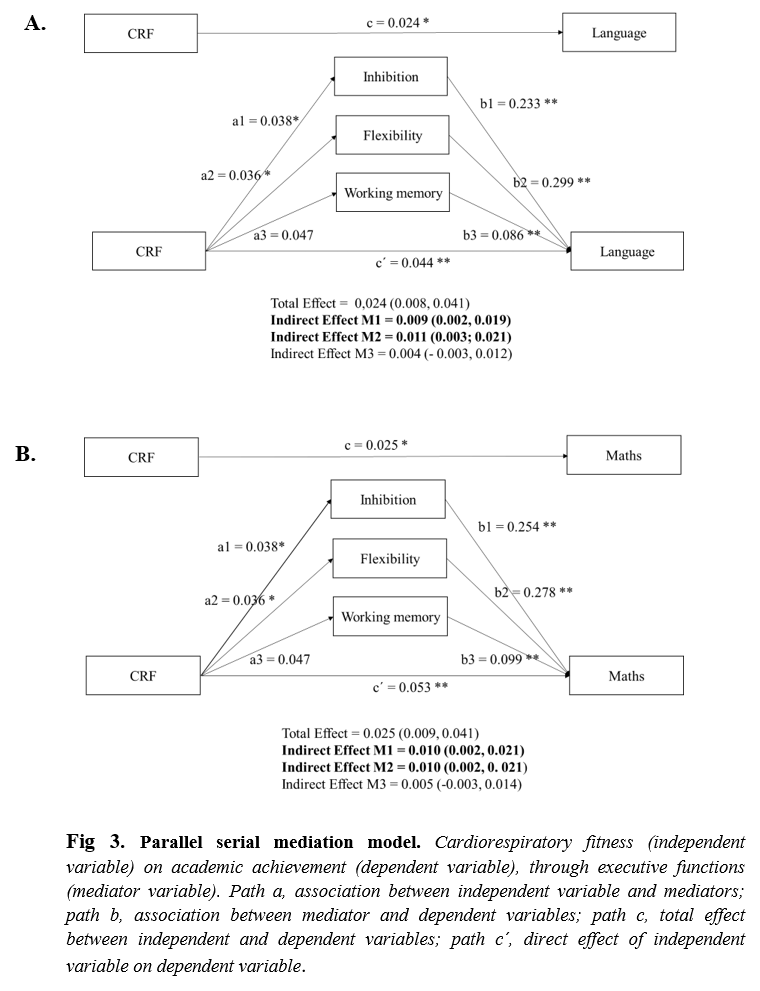

Supplement: S2 Fig — (TIF) [file pone.0231246.s002.tif]
