# Supplementary material for: Executive functions mediate the relationship between cardiorespiratory fitness and academic achievement in Spanish schoolchildren aged 8 to 11 years
Source: PLoS One. 2020 Apr 10;15(4):e0231246. doi: 10.1371/journal.pone.0231246 (PMC7147757; doi:10.1371/journal.pone.0231246)
Supplement: S1 Table — (DOCX) [file pone.0231246.s003.docx]

**S1.** Mean differences (ANCOVA) in academic achievement in language and mathematics by CRF categories, controlling for age and mother educational level.

|  |  | **CRF** | | | | |
| --- | --- | --- | --- | --- | --- | --- |
|  |  | **Lower Q** | **Middle Q** | **Higher Q** | **p** | **Partial eta sqared** |
| Total |  | (n = 465) | | | |  |
| Language | M1 | 6.70^MH^ (.151) | 7.28^H^ (109) | 7.81 (.150) | **<0.001** | 0.061 |
|  | M2 | 6.81^MH^ (.142) | 7.33 (.102) | 7.71 (.143) | **<0.001** | 0.047 |
| Mathematics | M1 | 6.42^MH^ (.156) | 7.05^H^ (.113) | 7.56 (.155) | **<0.001** | 0.059 |
|  | M2 | 6.52^MH^ (.147) | 7.10 (.105) | 7.43 (.147) | **<0.001** | 0.046 |
| Boys |  |  |  |  |  |  |
| Language | M1 | 6.79^H^ (.233) | 7.23 (.163) | 7.48 (.232) | 0.100 | 0.023 |
|  | M2 | 6.89 (.244) | 7.28 (.155) | 7.44 (.222) | 0.189 | 0.018 |
| Mathematics | M1 | 6.84^H^ (.226) | 7.20 (.159) | 7.55 (.226) | 0.087 | 0.025 |
|  | M2 | 6.90 (.213) | 7.24 (.148) | 7.49 (.212) | 0.143 | 0.020 |
| Girls |  |  |  |  |  |  |
| Language | M1 | 6.58^MH^ (.197) | 7.31^H^ (.146) | 8.10 (.194) | **<0.001** | 0.120 |
|  | M2 | 6.70^MH^ (.181) | 7.40^H^ (.148) | 7.96 (.183) | **0.000** | 0.098 |
| Mathematics | M1 | 6.02^MH^ (.215) | 6.92^H^ (.159) | 7.58 (.212) | **<0.001** | 0.108 |
|  | M2 | 6.13^MH^ (.203) | 6.98 (.150) | 7.42 (.205) | **0.000** | 0.087 |

Data are presented as marginal estimated mean ± standard error (SE). Abbreviations: CRF = cardiorespiratory fitness. Categories of CRF are lower Q (representing 1st quartile), middle Q (2nd and 3rd quartiles), and upper Q (4th quartile). The values in bold indicate statistical significance at p < 0.05. Model 1 (M1): analyses were adjusted for age and mother educational level. Model 2 (M2): analyses were adjusted for model 1 and CRF to executive function or executive function index to CRF categories. Superscript letters indicate statistical significance (p < 0.05) for the Bonferroni multiple comparison post-hoc test between the mean in the category and that indicated by superscript abbreviations (L = low, M = middle or H= high)
