# Supplementary material for: Executive functions mediate the relationship between cardiorespiratory fitness and academic achievement in Spanish schoolchildren aged 8 to 11 years
Source: PLoS One. 2020 Apr 10;15(4):e0231246. doi: 10.1371/journal.pone.0231246 (PMC7147757; doi:10.1371/journal.pone.0231246)
Supplement: S2 Table — (DOCX) [file pone.0231246.s004.docx]

**S2.** Mean differences (ANCOVA) in academic achievement in language and mathematics by inhibition, cognitive flexibility and working memory categories, controlling for age and mother educational level.

|  |  | **Inhibition** | | | | | | | **Cognitive flexibility** | | | | | | **Working memory** | | | | |
| --- | --- | --- | --- | --- | --- | --- | --- | --- | --- | --- | --- | --- | --- | --- | --- | --- | --- | --- | --- |
|  |  | **Lower Q** | **Middle Q** | **Higher Q** | | **p** | **Partial eta squared** | | **Lower Q** | **Middle Q** | **Higher Q** | **p** | **Partial eta squared** | | **Lower Q** | **Middle Q** | **Higher Q** | **p** | **Partial eta squared** |
| Total |  | (n = 539) | | | | | | |  | | | | | |  | | | | |
| Language | M1 | 6.69^MH^ (.142) | 7.37^H^ (.099) | 7.88 (.139) | **<0.001** | | | 0.070 | 6.52^MH^ (.139) | 7.44^H^ (.098) | 7.89 (.136) | **<0.001** | | 0.098 | 6.66^MH^ (.138) | 7.47^H^ (.098) | 7.75 (.144) | **<0.001** | 0.067 |
|  | M2 | 6.69^MH^ (.153) | 7.33^H^ (.106) | 7.84 (.151) | **<0.001** | | | 0.065 | 6.46^MH^ (.151) | 7.46 (.104) | 7.79 (.146) | **<0.001** | | 0.098 | 6.60^MH^ (.149) | 7.47 (.106) | 7.71 (.154) | **<0.001** | 0.072 |
| Mathematics | M1 | 6.34^MH^ (.149) | 7.06^H^ (.104) | 7.71 (.146) | **<0.001** | | | 0.082 | 6.25^MH^ (.148) | 7.15^H^ (.104) | 7.63 (.144) | **<0.001** | | 0.088 | 6.28^MH^ (.145) | 7.19^H^ (.103) | 7.58 (.151) | **<0.001** | 0.081 |
|  | M2 | 6.42^MH^ (.157) | 7.01^H^ (.109) | 7.69 (.155) | **<0.001** | | | 0.073 | 6.22^MH^ (.157) | 7.17 (.108) | 7.54 (.152) | **<0.001** | | 0.088 | 6.27^MH^ (.153) | 7.21^H^ (.109) | 7.49 (.159) | **<0.001** | 0.081 |
| Boys |  |  | |  |  | | |  |  | |  |  | |  |  | |  |  |  |
| Language | M1 | 6.15^MH^  (.209) | 7.26^H^  (.140) | 8.64  (.200) | **<0.001** | | | 0.163 | 6.20^MH^ (.213) | 7.32^H^ (.144) | 7.91 (.200) | **<0.001** | | 0.134 | 6.57^MH^ (.221) | 7.29 (.153) | 7.59 (.203) | **0.003** | 0.052 |
|  | M2 | 6.19^MH^ (.222) | 7.27^H^ (.150) | 8.13 (.216) | **<0.001** | | | 0.168 | 6.25^MH^ (.237) | 7.39 (.155) | 7.79 (.221) | **<0.001** | | 0.111 | 6.57^MH^ (.241) | 7.35 (.164) | 7.55 (.217) | **0.008** | 0.050 |
| Mathematics | M1 | 6.04^HM^ (.205) | 7.21^H^ (.137) | 8.10 (.196) | **<0.001** | | | 0.190 | 6.14^MH^ (.213) | 7.31 (.144) | 7.83 (.200) | **<0.001** | | 0.134 | 6.35^MH^ (.216) | 7.26 (.149) | 7.69 (.198) | **<0.001** | 0.088 |
|  | M2 | 6.17^HM^  (.215) | 7.23^H^  (.145) | 8.22  (.209) | **<0.001** | | | 0.192 | 6.27^MH^ (.232) | 7.36 (.152) | 7.80 (.217) | **<0.001** | | 0.112 | 6.34^MH^ (.231) | 7.40 (.157) | 7.65 (.208) | **<0.001** | 0.095 |
| Girls |  |  | |  |  | | |  |  | |  |  | |  |  | |  |  |  |
| Language | M1 | 7.11  (.189) | 7.47  (.136) | 7.73  (.190) | 0.068 | | | 0.022 | 6.76^MH^ (.184) | 7.56 (.132) | 7.88 (.183) | **<0.001** | | 0.077 | 6.71^MH^ (.175) | 7.63 (.127) | 7.98 (.206) | **<0.001** | 0.096 |
|  | M2 | 7.15 (.202) | 7.38 (.143) | 7.53 (.202) | 0.412 | | | 0.008 | 6.60^MH^ (.192) | 7.53 (.138) | 7.79 (.188) | **<0.001** | | 0.096 | 6.57^MH^ (.180) | 7.55 (.132) | 7.97 (.212) | **<0.001** | 0.121 |
| Mathematics | M1 | 6.56^H^ (.211) | 6.93 (.152) | 7.37 (.212) | **0.028** | | | 0.029 | 6.28^MH^ (.208) | 7.03 (.149) | 7.44 (.207) | **<0.001** | | 0.062 | 6.22^MH^ (.198) | 7.14 (.144) | 7.46 (.234) | **<0.001** | 0.074 |
|  | M2 | 6.63  (.223) | 6.83  (.159) | 7.20  (.223) | 0.194 | | | 0.016 | 6.10^MH^ (.215) | 7.03 (.154) | 7.32 (.211) | **<0.001** | | 0.080 | 6.16^MH^ (.204) | 7.05 (.150) | 7.38 (.242) | **<0.001** | 0.078 |

Data are presented as marginal estimated mean ± standard error (SE). Abbreviations: CRF = cardiorespiratory fitness. Categories of inhibition, cognitive flexibility and working memory are lower Q (representing 1st quartile), middle Q (2nd and 3rd quartiles), and upper Q (4th quartile). The values in bold indicate statistical significance at p < 0.05. Model 1 (M1): analyses were adjusted for age and mother educational level. Model 2 (M2): analyses were adjusted for model 1 and CRF to executive function or executive function index to CRF categories. Superscript letters indicate statistical significance (p < 0.05) for the Bonferroni multiple comparison post-hoc test between the mean in the category and that indicated by superscript abbreviations (L = low, M = middle or H = high).
