# Supplementary material for: Executive functions mediate the relationship between cardiorespiratory fitness and academic achievement in Spanish schoolchildren aged 8 to 11 years
Source: PLoS One. 2020 Apr 10;15(4):e0231246. doi: 10.1371/journal.pone.0231246 (PMC7147757; doi:10.1371/journal.pone.0231246)
Supplement: Attachment — Submitted filename: Prados review.docx [file pone.0231246.s005.docx]

Dear editor,

The paper focus on an important and emergent research area. However, in my opinion, deep changes on the manuscript should be considered prior to acceptance.

Abstract:

The authors should present some statistical data related to the main results, not only a p value.

The abstract readability can be improved by a scientific language edition (e.g: line 30 -31 change from “…showed significantly higher mean scores…” to “… higher scores…”).

Line 34: What means partially mediated?

The last sentence from the abstract is merely speculative and it is not related to the study conclusions.

How was the sampling process conducted? Please provide the inclusion and exclusion criteria.

Do the authors have formal assent from the children?

What is the time gap between academic performance data and CRF and cognitive functions assessments?

Line 107 Please change VO^2^max to VO_2max_

Line 109: What app was used to assess executive functions? Please change from ipad to tablet. Describe model and manufacturer in parentheses.

It is mandatory to the authors to provide more information about the measurements of academic performance.

Mother educational level is one of the main predictor of children academic performance. The authors should used this data as a controlling variable instead of a SES index, otherwise, the authors must present a better rationally for using this index.

Why partial correlations were not adjusted for SES?

Why the authors opt for using quartiles?

Did the authors calculated a priori sample size? What is the statistical power of the analyses?

I am not an expert in statistical, but I am not confident the authors made the right choices.

The authors should consider to present the data with the level of accuracy from the instrument used to measure the outcomes (e.g: there is no decimal places for age in years).

I suggest the authors to avoid the VO2 calculation using equation; they can simple use the last complete stage on shuttle run test.

What is the criteria for SES categorization?

Would be of value if the authors should use some data related to body composition in the analyses (at least a BMI could be presented).

The results section is hard to flow. Firstly, I believe the authors should rethink their statistical procedures. I strongly suggest the authors to avoid categorization and use continuous data.

The correlations may be presented in figures.

If the authors accept my suggestion and change the statistical approach, the discussion should be rewritten in the light of the new results.

For me the main limitations are: indirect assessment of CRF and academic performance; If academic performance and CRF are not assess in the same time point (as I am assuming), it could also be a strong limitation of the present investigation.
