# Supplementary material for: Executive functions mediate the relationship between cardiorespiratory fitness and academic achievement in Spanish schoolchildren aged 8 to 11 years
Source: PLoS One. 2020 Apr 10;15(4):e0231246. doi: 10.1371/journal.pone.0231246 (PMC7147757; doi:10.1371/journal.pone.0231246)
Supplement: Attachment — Submitted filename: Response_to_Reviewers.docx [file pone.0231246.s006.docx]

**Iratxe Puebla**

**Senior Managing Editor,**

**PLOS ONE**

Dear Editor,

Please find enclosed a revised version of our manuscript, “Executive functions as a mediator in the relationship between cardiorespiratory fitness and academic achievement in schoolchildren”. We would like to thank you for giving us the opportunity to revise and improve our manuscript. We also thank the reviewers for their thoughtful and constructive comments. We have considered all of the suggestions and have incorporated them into the revised manuscript. As a result, we believe our manuscript is stronger. An itemized point-by-point response to the reviewers’ comments is shown below.

Thank you for your attention in this matter.

Mairena Sánchez-López (corresponding author).

**Journal Requirements:**

1. There is some discrepancy between your manuscript and the details registered at www.clinicaltrials.gov, please clarify whether schoolchildren included were aged 8 to 11 years or 9 to 11 years, and whether the number of children enrolled in the study was 570 or 563.

**Authors’ response:** We apologize for the discrepancy. This variability in the data is due to the fact that the data registered at www.clinicaltrials.gov refer to the study design proposal. When we collected the data, we found some discrepancy regarding the age sample corresponding to the children who had birthdays in the last months of the year, before the first assessment. Additionally, the initial sample was composed of 570 children; however, some of the children did not meet the inclusion criteria, and 7 participants were removed.

2. Please amend either the title on the online submission form (via Edit Submission) or the title in the manuscript so that they are identical.

**Authors’ response:** Done. Thank you. In both the manuscript and the online submission form, the title is as follows:

*“Executive functions mediate the relationship between cardiorespiratory fitness and academic achievement in Spanish schoolchildren aged 8 to 11 years”.*

3. We note that you have included the phrase “data not shown” in your manuscript. Unfortunately, this does not meet our data sharing requirements. PLOS does not permit references to inaccessible data. We require that authors provide all relevant data within the paper, Supporting Information files, or in an acceptable, public repository. Please add a citation to support this phrase or upload the data that corresponds with these findings to a stable repository (such as Figshare or Dryad) and provide and URLs, DOIs, or accession numbers that may be used to access these data. Or, if the data are not a core part of the research being presented in your study, we ask that you remove the phrase that refers to these data.

**Authors’ response:** Thank you for the comment. We apologize for the mistake. As suggested, we have included these results as supporting information (Table S1 in supplementary material). Consequently, the following statement was changed:

“The results of the mediation analysis by gender showed that *after controlling for mother educational level, cognitive flexibility was not significant in girls*” (Page 14, lines 302-303).

* *Note for the reviewers: the references in brackets of pages and lines are referred to the Manuscript with track changes.*

**Response to Reviewer 1 Comments**

The investigators performed a cross-sectional analysis of the baseline data from a cluster-randomized controlled trial, aimed at assessing if the effectiveness of a physical activity program diminishes cardiometabolic risk and improves CRF, executive functioning and academic achievement in schoolchildren. Assuming the sample to have been adequately selected, the overall goal of examining physical fitness influences on academic achievement through executive functions appears to have been achieved. Although not a randomized comparison of low vs. high CRF, the descriptive nature of the study using correlational analysis, the ANCOVA with post hoc adjustment and the mediation analysis, is certainly interesting. The mediation model is well utilized, and the results are well explained. There are some minor concerns.

**Authors’ response:** Your comments and general involvement in the review process are highly appreciated. We have addressed all concerns, and we hope our revisions have improved the manuscript.

1. The manuscript should be edited for typos. For example, on line 26 of the abstract, the expression,’ Cross-sectional study including 539 schoolchildren, aged 8 to 11 years, from Cuenca, Spain.’ Is not a full sentence. On Table 3, one assumes the word ‘Hight’ should be ‘High’.

**Authors’ response:** Thank you for the comment. We apologize for the error. The paper has been sent again to a specialist service to correct mistakes and typos. The following changes have been made:

*“This was a cross-sectional study that included 570 schoolchildren, aged 8 to 11 years, from Cuenca, Spain”* (Page 2, lines 32).

Tables S1 and S2 (S1 and S2 Table Supplementary material).

2. On Table 1 the significant (p<0.05) p-values according to line 181 are in bold. Why are the p-values for the executive functions, TR congruent, incongruent and Total (0.245, 0.145 and 0.270, respectively) in bold type?

**Authors’ response:**  Thank you for the comment. We apologize for the mistake, which has been corrected (Table 1, page 10).

**Response to Reviewer 2 Comments**

The paper focus on an important and emergent research area. However, in my opinion, deep changes on the manuscript should be considered prior to acceptance.

**Authors’ response:** Your comments and general involvement in the review process are highly appreciated. We have addressed all concerns, and our revisions have improved the manuscript.

Abstract:

The authors should present some statistical data related to the main results, not only a p value.

**Authors’ response:** We appreciate this comment. As suggested, we have included the following information in the abstract section:

*“Overall, ANCOVA models controlling for age, gender and mother educational level showed higher scores in language and mathematics in children in higher categories of CRF, inhibition, cognitive flexibility and working memory than in children in lower categories. The effect sizes were moderate (p < 0.05, partial eta squared: from 0.05 to 0.12). Moreover,* *the mediation analysis showed that inhibition partially mediated the relationship between CRF and language (c’ = 0.058; IC = [0.005; 0.028]) and mathematics (c’ = 0.064; IC = [0.005; 0.030]) grades; similarly, cognitive flexibility mediated CRF’s relationship with language (c’ = 0.059; IC = [0.003; 0.028]) and with mathematics (c’ =0.066. ; IC = [0.003; 0.029]); however, a significant relationship remained. For working memory, mediation analysis showed no significant results (c′ = 0.92; IC= [-0.002;0.025] P > 0 .05 in language; c′ = 0.103; IC= [-0.002;0.029] P > 0.05 in mathematics). Mediation ranged from 13.38 % to 36 %*” (Page 2, lines 43-50).

The abstract readability can be improved by a scientific language edition (e.g: line 30 -31 change from “…showed significantly higher mean scores…” to “… higher scores…”).

**Authors’ response:** Thank you for the comment. As suggested, we have rephrased that sentence in the abstract section to improve readability:

*“ANCOVA models controlling for age, gender and mother educational level showed higher scores in language and mathematics in children (…)”* (Page 3, lines 37-38).

Moreover, full abstract has been improved following the comments and suggestions.

Line 34: What means partially mediated?

**Authors’ response:** According to Andrew F. Hayes (Hayes AF. Introduction to mediation, moderation, and conditional process analysis: a regression-based approach.: Guildford Press; 2018), partial mediation implies that the mechanism through the mediator variables (executive functions) does not entirely account for the association observed between the independent variable (CRF) and the dependent variable (academic achievement in language and mathematics), whereas complete mediation means that the association between CRF and academic achievement is entirely accounted for by the indirect mechanism. Partial mediation is the case in which the path from the independent variable to the dependent variable is reduced in absolute size but is still different from zero when the mediator variable is introduced in the model, whereas complete mediation occurs when the relationship between the independent variable and the dependent disappears after entering the mediator in the model, making the path c´ = 0.

In order to clarify this concern to the readers, the following changes have been made in the abstract section:

*“**(..) the mediation analysis showed that inhibition partially mediated the relationship between CRF and language (…) and mathematics (…); similarly, cognitive flexibility mediated CRF’s relationship with language (…) and with mathematics (…); however, a significant relationship remained”* (Page 2-3, lines 44-48)*.*

The last sentence from the abstract is merely speculative and it is not related to the study conclusions.

**Authors’ response:** Thank you for the comment. We agree regarding the inappropriateness of the sentence. Thus, the sentence has been modified as follows:

*“**The findings indicated that a significant proportion of the positive influence of CRF on academic achievement was mediated by improvements in inhibition and cognitive flexibility. Thus, improvements in CRF may contribute to increasing academic achievement not only through a direct mechanism but also through improvements in executive functions*” (Page 3 lines 52-55).

How was the sampling process conducted? Please provide the inclusion and exclusion criteria.

**Authors’ response:** Thank you for the thoughtful comment. As suggested, we have provided some detail on the sampling procedure as well as inclusion/exclusion criteria as follows:

**Methods**

This was a cross-sectional analysis of the baseline data from a cluster-randomized controlled trial (NCT03236337) aimed at assessing the effectiveness of a physical activity program (MOVI-daFit!) to diminish cardiometabolic risk and to improve CRF, executive functioning and academic achievement in schoolchildren. *The design, sampling procedures and methods for collecting the variable data have been described elsewhere (Martinez Vizcaíno et al Medicine (Baltimore). 2019 Mar;98(9):e1473)* (Page 5, lines 107-108).

***Participants***

This study included 570 schoolchildren, aged 8 to 11 years, attending 10 schools in Cuenca, Spain. *In these schools, all fourth- and fifth-grade children were invited to participate, except for those who met any of the following exclusion criteria: the children had Spanish learning difficulties; teachers or parents reported that the children had serious physical or mental disorders that could impede their participation in the program activities, or pediatricians reported that the children had chronic disorders such as heart disease, diabetes, or asthma that could prevent their participation in the program activities. Finally, our analysis included 563 participants for whom data on all study variables were collected* (Page 5, lines 111-116).

Do the authors have formal assent from the children?

**Authors’ response:** In this study, only the parents signed a written consent form for their children’s participation. However, every child was asked for their consent to participate before taking each test. Furthermore, children were informed of the characteristics of the study, and their opinion was considered in the design of the intervention. Before starting the MOVI intervention, the members of the research team visited the intervention school to facilitate a consensus among parents, teachers, and children on the types of games and modifications for the playground. During the study, through the data collection process, the children were asked to give us their consent to participate. We thought that this could increase compliance with the program.

We found this issue relevant. Thus, we have added the following sentence in the “Methods” section:

“*Every child was verbally asked for consent prior to the completion of each test*” (Page 6, lines 123-124).

What is the time gap between academic performance data and CRF and cognitive functions assessments?

**Authors’ response:** We thank you for your comment. To measure academic achievement, the final grades of the previous year were used. This was a measure that synthesizes students’ work for the whole year. In Spain, a final qualification is given to students as a result of a continuous evaluation for the entire academic year. This measure represents relatively stable behaviour or, in other words, students’ academic background. This grade is given in the month of June and represents students’ work for the whole year. We used that grade to assess academic achievement. We asked schools for this information during the months when the baseline study was performed, from September to October 2017. During these months, data were collected in two sessions two weeks apart. On the first visit, physical tests were performed, including CRF. On the second visit, participants completed a cognitive evaluation.

We agree that this measure of academic achievement could have several limitations. We discuss it below in another comment.

Line 107 Please change VO2max to VO_2max_

**Authors’ response:** Done. Thank you.

Line 109: What app was used to assess executive functions? Please change from Ipad to tablet. Describe model and manufacturer in parentheses.

**Authors’ response:** Thank you. As suggested, we have included a description of the app used for assessing executive functions and replaced Ipad with tablet in the methods section as follows:

“NIH Toolbox (NIH Toolbox in Spanish, v. 1.8)” (page 7, lines 151).

“All measurements were performed (…) using a tablet (iPad Pro, Apple, Inc.) (page 7, lines 152-153)”.

It is mandatory to the authors to provide more information about the measurements of academic performance.

**Authors’ response:** Thank you for the suggestion. We have modified the text as follows:

“*Academic achievement was measured with data provided by the schools. These data consisted of children’s final grades in language and mathematics from the previous year (2016/17, 3rd and 4th grades). In Spain, academic achievement is measured on a 0 to 10 scale, with 10 being the maximum grade. Final grades represent the student´s work in an entire academic year, as assessed by the teacher in a continuous evaluation process”* (Page 6-7, lines 139-143)*.*

Mother educational level is one of the main predictor of children academic performance. The authors should used this data as a controlling variable instead of a SES index, otherwise, the authors must present a better rationally for using this index.

**Authors’ response:** We sincerely appreciate the good suggestion. We have repeated all analyses using mother educational level as a control variable.

Changes can be observed in the methods section (Page 8, 183-191; Page 10, 24’), Table 1 (Page 11); Table 2 (Page 13); Table S1 and S2 (S1 and S2 Table Supplementary material) and Table 3 (Page 18).

Why partial correlations were not adjusted for SES?

**Authors’ response**: This comment seems appropriate; thus, mother educational level has been included as a covariate in Table 2 (Page 13).

Why the authors opt for using quartiles?

**Authors’ response:** We appreciate the comment. Although it has been proposed to use cut offs for dichotomizing CRF levels (please see Ruiz JR et al Br J Sports Med. 2016;50:1451-1458), the aim of categorizing variables measured on an interval scale was to test differences between low, middle and high fitness levels. For this, several authors have used quartiles as a standard procedure (e.g., Álvarez-Bueno et al., 2019. Cardiorespiratory fitness as a mediator of the relationship between birth weight and cognition in school children. *Psychology research and behaviour management,* 12, 255), since there is no universally accepted procedure for this aim.

To clarify the aims of these ANCOVA procedures, which may be unclear, the following sentences were included in the methods section:

*“**Because there are no universally accepted cut offs for categorizing low, middle and high CRF and executive performance, we used quartiles for this aim as follows: low (Q1), middle (Q1-Q2) and high (Q4). Using these categories, ANCOVA models were estimated to examine differences in academic achievement (language and mathematics) by CRF and executive function performance (inhibition, cognitive flexibility and working memory). These ANCOVA models were also used to examine the potential confounding or mediating role of executive functions in the relationship between CRF and academic achievement”* (Page 9, lines 203-209)*.*

*“**In these models, language and mathematics grades were used as dependent variables and CRF, inhibition, cognitive flexibility and working memory categories as fixed factors*” (Page 9, lines 209-211).

“*For the rest of the analyses, continuous variables were used”* (Page 9, lines 217-218).

Did the authors calculated a priori sample size? What is the statistical power of the analyses? I am not an expert in statistical, but I am not confident the authors made the right choices.

**Authors’ response:** The sample size was estimated not for mediation analyses but for a cluster randomized trial belonging to a representative sample from Cuenca, Spain. A new reference has been included in which the study protocol is described in more depth.

However, the precision of the confidence intervals, as well as the recommended sample size for the mediation analysis (Fritz MS, MacKinnon DP. Required sample size to detect the mediated effect. Psychol Sci. 2012; 18(3):233-239), indicate that the sample size is large enough for the analyses included in this manuscript.

The authors should consider to present the data with the level of accuracy from the instrument used to measure the outcomes (e.g: there is no decimal places for age in years).

**Authors’ response:** Thank you for the comment. As suggested, since age was obtained in months and the cut offs for classifying weight status in children are estimated for age in months (Cole TJ, Lobstein T. Extended international (IOTF) body mass index cut-offs for thinness, overweight and obesity. Pediatr Obes. 2012; 7(4): 284-94), we have included age in months in the tables and analyses (Table 1, Page 10).

I suggest the authors to avoid the VO2 calculation using equation; they can simple use the last complete stage on shuttle run test.

**Authors’ response:** Thank you for the comment. We assessed both variables before deciding to use VO_2max_. The main reason for choosing this variable was that VO_2max_ is the most continuous variable, whereas stage is a variable that represents the stage reached. We found this kind of variable more adapted to the procedures we were planning to perform. Another reason to choose VO_2max_ is that this is an indicator that considers weight (mL/kg/min). In a population with a considerable prevalence of overweight/obesity (28%), this is an important fact to consider. However, all analyses were performed with both variables with similar results. We also checked previous research in which this variable was used for analyses (e.g., Aadland et al., 2017; Castro-Piñero et al., 2017; Álvarez-Bueno et al., 2019; Scott et al. 2016).

What is the criteria for SES categorization?

**Authors’ response:** Following your suggestions, we used mother educational level instead of SES as a control variable in all analyses. The SES variable has been relegated to descriptive purposes in Table 1. For this aim, we used an index developed by the Spanish Society of Epidemiology and the Spanish Society of Family and Community Medicine following criteria proposed by Goldthorpe that are commonly used in UK (please see Domingo‐Salvany A et al. Proposal for a social class measure. Working Group of the Spanish Society of Epidemiology and the Spanish Society of Family and Community Medicine. *Aten Primaria*. 2000; 25: 350‐ 363). Thus, we have included this information in the manuscript as follows:

*“To asses socioeconomic status, we surveyed parents using the scale proposed by the Spanish Society of Epidemiology [22], with items related to employment and the highest level of education obtained by both the father and mother. Five SES categories were obtained: lower, lower middle, middle, upper middle and upper. Because most authors have reported that mother education level is a strong predictor of children’s academic achievement, we obtained this variable using items related to the highest level of education obtained by the mother from this questionnaire. We thus obtained a five-category index: no reading/no studies, elementary studies, secondary studies, high school and university studies*” (Page 8, lines 183-191).

Would be of value if the authors should use some data related to body composition in the analyses (at least a BMI could be presented).

**Authors’ response:** Thank you for the recommendation. We have included BMI in Table 1 (Page 11) and Table 2 (Pages 13). As the association between BMI and the dependent variables was not significant, we decided not to include this variable as a covariate in the subsequent analyses.

The results section is hard to flow. Firstly, I believe the authors should rethink their statistical procedures. I strongly suggest the authors to avoid categorization and use continuous data.

**Authors’ response:** Thank you for the comment. According to your suggestion, our analyses incorporated the variables at the interval scale for testing the association between academic achievement, cognitive performance and cardiorespiratory fitness and for estimating the regression coefficients included in the mediation analyses. However, to provide a more comprehensive analysis, we tested differences in the means of the academic achievement variables by executive functions and physical fitness categories, which was our first objective (to analyse whether there are differences in academic achievement in language or mathematics between low- and high-fit children and between low- and high-executive-functioning children). Likewise, we examined the potential confounder or mediator role of executive functions by using ANCOVA models in which cardiorespiratory fitness was categorized as low, middle and high; this is a common practice and can provide readers a comprehensive view and complement interval-scale analyses of changes in the relationship between independent and dependent variables.

This procedure has been performed in previous studies (e.g., Diez Fernandez et al. Diabetes Care. 2014;37(3):855-62).

However, we agree with you that this section could be difficult to follow. Thus, we have replaced the ANCOVA with figures (Fig 1 and Fig 2) in the main text. The table is included as supplementary material (S1 and S2), and the ANCOVA procedures are described in the methods section as follows:

*‘**Because there are no universally accepted cut offs for categorizing low, middle and high CRF and executive performance, we used quartiles for this aim as follows: low (Q1), middle (Q1-Q2) and high (Q4). Using these categories, ANCOVA models were estimated to examine differences in academic achievement (language and mathematics) by CRF and executive function performance (inhibition, cognitive flexibility and working memory). These ANCOVA models were also used to examine the potential confounding or mediating role of executive functions in the relationship between CRF and academic achievement. In these models, language and mathematics grades were used as dependent variables and weight status, CRF, inhibition, cognitive flexibility and working memory categories as fixed factors. In these models, language and mathematics were used as dependent variables and CRF, inhibition, cognitive flexibility and working memory categories as fixed factors, controlling for age and mother educational level (Model 1). Further adjustment for the executive function index or CRF, depending on the fixed factor, was included in Model 2. Pairwise post hoc hypotheses were tested using the Bonferroni correction for multiple comparisons.* *Effect size partial eta^2^ for ANCOVA test was calculated. Indicates small effects values of 0.01, intermediate effects 0.06 and strong effects >0.014 (**Cohen J. Statistical power analysis for the behavioral sciences. New York: Academic Press 1988, pp 280-287). These ANCOVA models were estimated for the entire sample and stratified by gender. For the rest of the analyses, continuous variables were used’* (Page 9, lines 203-218).

The correlations may be presented in figures.

**Authors’ response:** We appreciate your comment. We have added the following correlation matrix plot, performed in Python, to the supplementary material (S1 Fig).

If the authors accept my suggestion and change the statistical approach, the discussion should be rewritten in the light of the new results.

**Authors’ response:** Thank you for the comment. As suggested, we have added the following sentences to the Discussion section:

*“Our study suggested that* the association between CRF and academic achievement in mathematics and language was partially mediated by inhibition and cognitive flexibility, even after controlling for age *and mother educational level”* (Page 19, lines 358).

*“Our results indicated that the mediator effect of executive function between CRF and academic achievement was approximately 13.4 to 36 %”* (Page 20, lines 396-397).

*“However, mediation analyses revealed a stronger mediating role of executive functions in the relationship between fitness and academic achievement in mathematics in boys than in girls after controlling for mother educational level. This might be due to the higher influence of mother educational level on girls than on boys”* (Page 21, lines 415-418).

For me the main limitations are: indirect assessment of CRF and academic performance; If academic performance and CRF are not assess in the same time point (as I am assuming), it could also be a strong limitation of the present investigation.

**Authors’ response:** Your comment is greatly appreciated. We have added the following sentences to “*Strengths and limitations”* in the Discussion section:

*“**Another limitation of the study was that both CRF and academic performance were not assessed simultaneously, and they were assessed using indirect procedures, i.e., the 20-meter shuttle run test and teacher evaluations. We should consider that academic achievement is the result of a long-term learning process, and the cross-sectional evaluation actually implies the final outcome of a 1-year learning follow up. Regarding the validity of the 20-meter shuttle run test to estimate CRF, this is the recommended test in the ALPHA battery, since the direct measurement of VO_2_max in population-based studies is unfeasible”* (Page 21, lines 425-431)*.*

**Response to Reviewer 3 Comments**

Executive functions as a mediator in the relationship between cardiorespiratory fitness and academic achievement in schoolchildren.

Comments

Title: can the title be changed to a declarative statement (e.g. Executive functions mediate the relationship between…) and better specify the study population (add country)?

**Authors’ response:** We appreciate the suggestion, and the following change has been made:

*“**Executive functions mediate the relationship between cardiorespiratory fitness and academic achievement in Spanish schoolchildren aged 8 to 11 years”* (Page 1-2)*.*

Abstract:

“It has been hypothesized that” or previous researched have actually studied this relationship?

**Authors’ response:** Thank you for the comment. We have modified the sentence as follows:

*“**Previous research has studied the influence of physical fitness on academic achievement through executive functions”* (Page 2, lines 25-26).

“the associations between cardiorespiratory fitness (CRF) and the executive function domains with academic achievement and…” is unclear. Associations between A and B with C? Do you mean interrelationships (e.g. A and B, A and C, B and C)?

**Authors’ response:** Thank you for the comment. As suggested, we have modified sentence as follows:

“*This study aimed to analyze how cardiorespiratory fitness (CRF) and executive functions are associated with academic achievement*” (Page 2, lines 28-29)

Line 26 – Add “This” before cross-sectional.

**Authors’ response:** Done. Thank you for the comment.

Can you add a little more detail on the measurement of CRF and EF? Just add shuttle-run and NIH Toolbox.

**Authors’ response:** Thank you for the comment. We now include more information on the measures of CRF and EF in the abstract, which has been modified as follows:

*“CRF (20-meter shuttle run test) and executive functions (inhibition, cognitive flexibility and working memory, NIH Toolbox battery in Spanish, v 1.8; iPad Pro, Apple, Inc.) were measured”* (Page 2, lines 34-36)*.*

Can you add effect sizes to the abstract?

**Authors’ response:** Thank you. As suggested, we have included ES information in the abstract, and in table S1 (supplementary material), partial eta squared has been included:

“*…children in higher categories of CRF, inhibition, cognitive flexibility and working memory than in children in lower categories. The* *effect sizes were small to medium (p < 0.05, partial eta squared: from 0.05 to 0.12)* (Page 2, lines 43-44).

And we have added the following sentence (in Data analyses section, page 9, lines 215-216):

“…*Effect size partial eta^2^ for ANCOVA test was calculated. Values of 0.01, 0.06, and 0.14 to indicate small, medium, or large effects* (Cohen J. Statistical power analysis for the behavioral sciences. New York: Academic Press 1988, pp 280-287)”.

Line 36 – this sentence errs on the causal side and; would reword to state that children in this study with higher CRF and EF had higher math and language scores. It’s just an association in this sample.

**Authors’ response:** Thank you for the comment. We have rephrased the sentence to avoid establishing a causal relation, as follows:

“*Children in higher categories of both CRF and executive function showed higher grades in mathematics and language”* (Page 3, lines 51-52)*.*

Line 38 – mediated (past tense); otherwise it sounds like a population inference

**Authors’ response:** Thank you for the suggestion. The error has been modified.

Introduction

You may also want to cite

• Scott et al. 2016, Med Sci Sport Exer – Cardiorespiratory fitness is associated with better executive function in young women

• Scott et al. 2016, Journal of Nutrition – Combined iron deficiency and low aerobic fitness doubly burden academic performance among women attending university (see fig 3 for mediation)

**Authors’ response:** The comment is greatly appreciated. We found that those references support the relationship between CRF and cognition. Thus, we have included the references proposed by the reviewer in the introduction section (Page 4, lines 69, 76) and included references 6 and 11 in the bibliography.

Line 73 – same problem as in abstract: Associations between A and B with C?

**Authors’ response:** Thank you for the comment. We have reformulated the sentence as follows:

*“the association between CRF and academic achievement (in language and mathematics) and the association between the three executive function domains and academic achievement”* (Page 5, lines 92-93).

Methods

Line 98 – “in standardized conditions”? Do you mean “following standard procedures”? Was this done by nurses or the researchers?

**Authors’ response:** Thank you for the comment. We have rephrased the paragraph as follows:

*“The research was carried out by a multidisciplinary team composed of nurses, psychologists, teachers and physical activity experts. Each test of the study was carried out by the most appropriate team members (e.g., anthropometric tests were carried out by nurses, cognitive tests by psychologists**, physical activity tests by sports science educators) (*Page 6, lines 125-128).

20 trials on the flanker task does not seem sufficient. Were multiple blocks performed? If you used only 20 trials, can you cite other studies to support that this is acceptable?

**Authors’ response:** Thank you for the comment. In our study, to measure cognitive inhibition, we used the NIH Flanker Inhibitory Control and Attention Test. This is a test included in a wider battery of tests aimed at assessing cognitive function in people aged 3-85 years. The validation process has been described elsewhere (Weintraub S, Dikmen SS, Heaton RK, Tulsky DS, Zelazo PD, Bauer PJ, et al. Cognition assessment using the NIH Toolbox. Neurology. 2013;80(11 Suppl 3):S54-64.), and the authors conclude that 20 trials is appropriate. In children aged 3-7, two blocks of trials are performed. The first one consists of a set of 20 fishes, and if a participant scores ≥ 90% on the fish stimuli, 20 additional trials with arrows are presented. Before the 20-trial block, 4 practice trials are performed in all tests. After checking validation and information about the NIH Toolbox, we found that the NIH Toolbox was a reliable measure for cognition.

To clarify this concern, we have added the following references supporting the validity and suitability of this test:

Zelazo PD, Anderson JE, Richler J, Wallner-Allen K, Beaumont JL, Weintraub S. II. NIH Toolbox cognition battery (cb): measuring executive function and attention: NIH Toolbox cognition battery (CB). *Monographs of the Society for Research in Child Development*. 2013;78(4):16-33

Brito NH, et al. Associations between cortical thickness and neurocognitive skills during childhood vary by family socioeconomic factors. *Brain and cognition.* 2017, vol. 116, p. 54-62.

Akshoomoff N, Brown T, Bakeman R, Hagler D. Developmental differentiation of executive functions on the NIH Toolbox Cognition Battery. *Neuropsychology*. 2018. 32(7), 777-7783.

Calderon J, Bellinger DC, Hartigan C, Lord A, Stopp C, Wypij D, Newburger JW. Improving neurodevelopmental outcomes in children with congenital heart disease: protocol for a randomised controlled trial of working memory training. 2019. BMJ open, 9(2).

Would it not be useful to treat accuracy and speed as separate variables? Were children with <80% accuracy dropped from the analysis?

**Authors’ response:** Thanks for the comment. In order to clarify this concern, we have included the following information about the convenience or not of jointly assessing accuracy and speed in the limitation section:

*Because some authors recommend the measurement of accuracy and reaction time as separate variables, the assessment of these cognitive abilities as an unique score could be seen as a limitation; however, the NIH Toolbox´s total score has been used and validated in several studies and allows the avoidance of a ceiling effect in accuracy, which is typically achieved by the most of participants of this age”* (Page 22, lines 436-440).

*“**For children scoring < 80%, a total score considering accuracy was performed”* (Page 7, 164-165; 173-174).

Line 132 – what are aliments? Food items?

**Authors’ response:** Yes, we mean “food items”. We apologize for the confusion, and this text has been corrected. Thank you for the comment.

Please provide more information on the SES index. What were the education and employment categories and how were these used to construct the SES index? You say highest level of education in their family (all family members) and then either father or mother. Which was it? Too bad you didn’t ask about their income.

**Authors’ response:** Thank you for the thoughtful comment. Because most authors recognize not a whole SES assessment but mother education level as the main predictor of academic achievement, we have used SES categories for the description of the study sample characteristics and mother education level (gathered from the items included in the SES measurement) as control variables. We used an index developed by the Spanish Society of Epidemiology and the Spanish Society of Family and Community Medicine, following criteria proposed by Goldthorpe that are commonly used in the UK (please see Domingo‐Salvany A et al. Proposal for a social class measure. Working Group of the Spanish Society of Epidemiology and the Spanish Society of Family and Community Medicine. Aten Primaria. 2000; 25: 350‐ 363), as used in previous studies.

This information has been included in the methods section as follows:

*“To asses socioeconomic status, we surveyed parents using the scale proposed by the Spanish Society of Epidemiology [22], with items related to employment and the highest level of education obtained by both the father and mother. Five SES categories were obtained: lower, lower middle, middle, upper middle and upper. Because most authors have reported that mother education level is a strong predictor of children’s academic achievement, we obtained this variable using items related to the highest level of education obtained by the mother from this questionnaire. We thus obtained a five-category index: no reading/no studies, elementary studies, secondary studies, high school and university studies”* (Page 8, 183-191).

The model descriptions are unclear to me. All of your variables are continuous, so why not just use OLS regressions with multiple predictors? “We estimated ANCOVA models to test mean differences in language and mathematics by categories of CRF and executive function domain, controlling for age, pubertal status and SES (Model 1); further adjustment for the executive function index or CRF, depending on the fixed factor, was included in Model 2.”

**Authors’ response:** Your concern is valid. To address it, we have included a paragraph in the introduction section justifying the mediation analysis.

*“Health studies commonly control for confounding or mediator variables using multivariate methods such as multiple linear regression, logistic regression, or ANCOVA, depending on the study aims and/or whether the dependent variable is quantitative or categorical. Mediation analysis is a statistical procedure suitable for clarifying whether a third variable underlies the relationship between two variables and determining the extent to which this relationship can be modified, mediated, or confounded by this third variable (Baron RM, Kenny DA. The moderator-mediator variable distinction in social psychological research: conceptual, strategic, and statistical considerations. J Pers Soc Psychol 1986;51:1173–1182). We consider that a mediation effect occurs when a significant proportion of the influence of the independent variable on the dependent variable takes place through a third variable (mediator)”* (Page 5 95-102)*.*

Does adjusting for pubertal status (I think you mean stage; status implies a dummy variable, i.e. pre/post pubertal) really add much? You already adjust for age, which I would assume is colinear with pubertal status, though I could be wrong.

**Authors’ response:** We apologize for the mistake, which has been corrected. We mean stage, not “status”.

According to your suggestion, we have repeated some analyses, and we find reasons to remove pubertal stage from the adjustment. First, most children in the sample had very low pubertal stage development (86% presented a pubertal stage <=2); moreover, this variable was closely associated with age. Thus, as the reviewer recommends, pubertal stage has been removed as a control variable. These changes can be observed in the methods section (Page 8, lines 194-196), Table 2 (Page 13), Table 3 (Page 18) and Table S1 and S2 (Supplementary material).

Results

Table 1

-add BMI

**Authors’ response:** Done. BMI is now included in Table 1 (Page 11) and Table 2 (Page 13). Thanks for the comment.

-add % falling into fitness categories based on CRF? VO_2max_ of 46 seems fairly fit, but I don’t know what the ACSM cutoffs are in this age group. Need to get a sense of whether sample is fit or unfit.

**Authors’ response:** Thanks for the comment.

CRF categories were made attending to quartiles in our sample because at this age, there are no ACSM categories. Some authors have proposed points that could indicate a “red flag” of cardiovascular disease risk (e.g., Ruiz et al., 2015).

As suggested, to provide the readers an estimation of the children’s fitness levels, we have included the VO_2max_ levels corresponding to each category in Table 1 (Page 11).

-need units for EF indicators. I assume accuracy is % incorrect responses? This is typically expressed as proportion correct.

**Authors’ response:** Thank you for the comment. Accuracy refers to total correct responses. Reaction time is time in milliseconds. The total score is a score that takes into account accuracy and time reaction, calculated by the app using the previously explained algorithm. It provides a score from 0-10.

To improve understandability, we have included the appropriate units of EF indicators in Table 1 (Page 11).

Table 3 is very hard to follow. I suggest moving this to supplement and creating a figure or figures for the main paper.

**Authors’ response:** Thank you for the comment. Table 3 has been moved to the supplementary material (S1 and S2 Tables), and a figure synthesizing the differences in academic achievement between children in lower and higher categories of CRF and EF has been included (Fig 1 and Fig 2).

Discussion – can you comment on what may mediate the other 70-85% of the relationship between CRF and language/math that is not explained by executive function, age, pubertal status, and SES?

**Authors’ response:** Thank you for the recommendation. As suggested, we have included some sentences addressing this concern in the discussion section as follows:

*“**Our results indicated that the moderator effect of EF between CRF and academic achievement was approximately 13.4 to 36 %. Several other fitness-related factors could influence academic achievement, such as motor skills. Two studies showed that motor skills, rather than aerobic fitness or physical activity, had the strongest association with executive functions and academic performance [33,34]. A review study [35] proposed that both CRF and motor skills were related to academic performance and that the influence of both could affect cognition and academic performance in different ways. Moreover, a mediation study showed that motor coordination—not cardiovascular endurance or muscular strength—was the only motor ability that accounted for a significant percentage of the variance in academic achievement [36]. Other variables that have been demonstrated to be related to academic achievement, possibly influencing the relationship between CRF and academic achievement, are physical activity and general health improved by cardiorespiratory fitness (Marques, Santos, Hillman & Sardinha, 2018)”* (Page 19, lines 396-406).
