# Supplementary material for: Executive functions mediate the relationship between cardiorespiratory fitness and academic achievement in Spanish schoolchildren aged 8 to 11 years
Source: PLoS One. 2020 Apr 10;15(4):e0231246. doi: 10.1371/journal.pone.0231246 (PMC7147757; doi:10.1371/journal.pone.0231246)
Supplement: Attachment — Submitted filename: renamed_390ab.doc [file pone.0231246.s007.doc]

**COMMENTS – Manuscript D-19-14578R1**

Title: “Executive functions mediate the relationship between cardiorespiratory fitness and academic achievement in Spanish schoolchildren aged 8 to 11 years”

**Important note: This review pertains only to ‘statistical aspects’ of the study and so ‘clinical aspects’ [like medical importance, relevance of the study, ‘clinical significance and implication(s)’ of the whole study, etc.] are to be evaluated [should be assessed] separately/independently.**

Please note that it is preferable [refer to item 1b of CONSORT checklist 2010: Structured summary of trial design, methods, results, and conclusions; though article type is ‘Research Article’] to divide the ABSTRACT with small sections like ‘Objective(s)’, ‘Methods’, ‘Results’, ‘Conclusions’, etc. which is a accepted practice of most good/standard journals [including PLOS]. Your ABSTRACT is well drafted but assay type.

This being a cross-sectional study, there are few ‘design’ issues involved, regarding which, I am sure, authors are well aware [clear as it is mentioned in ‘*Strengths and limitations*’ section later]. All sorts of biases [more likely ‘selection’ biases] are common occurrence in such cross-sectional studies. A cross-sectional study is very easy to conduct but is of very limited value [in no way any cross-sectional study will yield confirmatory conclusion mainly because of absence of temporal sequence]. Since any cross-sectional study is considered as a low level of [scientific] evidence, such design used only if that is what is possible [that is the only possibility]. {This point is raised here particularly in the backdrop of certainty needed in finding answer(s) to posed question(s)}.

Is the reference number 18 [Baron RM, Kenny DA. The Moderator-Mediator Variable Distinction in Social Psychological Research: Conceptual, Strategic, and Statistical Considerations] complete? (this question is raised because this reference is of vital importance, in my opinion). Although this study is off-shoot of another study [MOVI-daFIT! Intervention: Rationale and design of a cluster randomized controlled trial testing the effects on improving adiposity, cognition, and subclinical atherosclerosis by increasing cardiorespiratory fitness in children], it seems to be methodologically well conducted/executed one. My only query is : why line numbers are not given for ‘discussion’ section?
