# Supplementary material for: Executive functions mediate the relationship between cardiorespiratory fitness and academic achievement in Spanish schoolchildren aged 8 to 11 years
Source: PLoS One. 2020 Apr 10;15(4):e0231246. doi: 10.1371/journal.pone.0231246 (PMC7147757; doi:10.1371/journal.pone.0231246)
Supplement: Attachment — Submitted filename: 4. Response to reviewers.docx [file pone.0231246.s008.docx]

Dear Editor,

Please find enclosed a revised version of our manuscript, “Executive functions as a mediator in the relationship between cardiorespiratory fitness and academic achievement in schoolchildren” (PONE-D-19-14578R1). We would like to thank you for giving us the opportunity to revise and improve our manuscript; we also thank the reviewers for the thoughtful and constructive comments. We have considered all the suggestions and have incorporated them into the revised manuscript, and as a result, we believe our manuscript is stronger. An itemized point-by-point response to the reviewers’ comments is shown below.

Thank you for your attention in this matter.

Mairena Sánchez-López (corresponding author).

**Response to reviewer 4 Comments**

Important note: This review pertains only to ‘statistical aspects’ of the study and so ‘clinical aspects’ [like medical importance, relevance of the study, ‘clinical significance and implication(s)’ of the whole study, etc.] are to be evaluated [should be assessed] separately/independently.

Please note that it is preferable [refer to item 1b of CONSORT checklist 2010: Structured summary of trial design, methods, results, and conclusions; though article type is ‘Research Article’] to divide the ABSTRACT with small sections like ‘Objective(s)’, ‘Methods’, ‘Results’, ‘Conclusions’, etc. which is the accepted practice of most good/standard journals [including PLOS]. Your ABSTRACT is well drafted but assay type.

**Authors response:** We sincerely appreciate the reviewer´s comment; thus, as suggested, we have structured the abstract (see Page 2, Abstract section).

This being a cross-sectional study, there are few ‘design’ issues involved, regarding which, I am sure, authors are well aware [clear as it is mentioned in ‘Strengths and limitations’ section later]. All sorts of biases [more likely ‘selection’ biases] are common occurrence in such cross-sectional studies. A cross-sectional study is easy to conduct but generally is of very limited value [since in no way any cross-sectional study will yield confirmatory conclusion(s) mainly because of absence of temporal sequence]. Since any cross-sectional study is considered as a low level of [scientific] evidence, such design is used only if that is what is possible [that is the only possibility]. {This point is raised here particularly in the backdrop of certainty needed in finding answer(s) to posed question(s)}.

**Authors response:** Thank you for the comment. Although temporal ambiguity is certainly a core limitation to stablish causality in the relationship analyses, our mediation models, as some authors such as Stillman et al. ^1^, support the usefulness of these statistical mediation models to test plausibility of causal models, when experimental manipulation (the gold standard for assessing causality) is not available. Moreover, although this temporal limitation cannot be ruled out, it seems more plausible, according to the existing literature ^2–4^, that higher cardiorespiratory fitness causes better academic achievement, and not the opposite.

Therefore, as suggested, we have rephrased some sentences related to the reviewer´s concern:

“*Thus, this study supports the hypothesis that improvements in CRF may contribute to increasing academic achievement not only through a direct mechanism but also through improvements in executive functions*” (Page 3, line 44).

“A*lthough previous studies support a directional relationship between physical activity and fitness to academic achievement,* *the cross-sectional nature of our analyses prevented us from making cause–effect inferences, since temporal ambiguity bias cannot be completely ruled out*” (Page 17, lines 369-372).

1.	Stillman, C. M., Cohen, J., Lehman, M. E. & Erickson, K. I. Mediators of Physical Activity on Neurocognitive Function: A Review at Multiple Levels of Analysis. *Frontiers in Human Neuroscience* **10**, (2016).

2.	Álvarez-Bueno, C. *et al.* The Effect of Physical Activity Interventions on Children’s Cognition and Metacognition: A Systematic Review and Meta-Analysis. *Journal of the American Academy of Child & Adolescent Psychiatry* **56**, 729–738 (2017).

3.	Donnelly, J. E. *et al.* Physical Activity, Fitness, Cognitive Function, and Academic Achievement in Children: A Systematic Review. *Med Sci Sports Exerc* **48**, 1197–1222 (2016).

4.	Hillman, C. H. *et al.* Effects of the FITKids randomized controlled trial on executive control and brain function. *Pediatrics* **134**, e1063-1071 (2014).

Is the reference number 18 [Baron RM, Kenny DA. The Moderator-Mediator Variable Distinction in Social Psychological Research: Conceptual, Strategic, and Statistical Considerations] complete? (this question is raised because this reference is of vital importance, in my opinion). Although this study is off-shoot of another study [MOVI-daFIT! Intervention: Rationale and design of a cluster randomized controlled trial testing the effects on improving adiposity, cognition, and subclinical atherosclerosis by increasing cardiorespiratory fitness in children], it seems to be methodologically well conducted/executed one. My only query is: why line numbers are not given for ‘discussion’ section?

**Authors response:** Thank you, we apologize for the inconvenience. Both typos have been corrected (please, see pages 15-19, in discussion section and this reference corrected now reference 37, page 24, lines 521-524).

37. Baron, R. M. & Kenny, D. A. The Moderator-Mediator Variable Distinction in Social Psychological Research: Conceptual, Strategic, and Statistical Considerations. search. *Journal of Personality and Social Psychology* **51** 1173-1182 (1986).

**Response to reviewer 4 Comments**

This manuscript presents baseline data from a randomized controlled trial and examined the mediating role of executive functioning in the relationship between cardiovascular fitness and academic achievement. This study is well powered and answers an interesting research question. Further thoughts are presented below:

Major Issues:

There is little to no background given on why males and females are examined separately. This should be expanded on in the introduction since it is such a large piece of the analytic strategy.

**Authors response:** Thank you. The reviewer’s comments and, in general, his/her involvement in the review process are highly appreciated. In order to duly justify the need to present the results separated by gender, we have modified the paragraph as follows:

*“Additionally, no study has provided results separately by gender, even though gender differences in the association between CRF and academic achievement have been reported [17]. Moreover, differential brain maturation and executive function development between boys and girls have been reported [18]” (Page 5, lines 77-80).*

The results and data analysis are extremely unclear. Was a multiple mediation analysis conducted or were separate mediation analyses conducted for each potential mediator and each DV? It appears like separate mediation analyses were conducted. It would seem like a better analytic approach would be to examine a multiple mediation model and determine which EF domains are mediating the relationship instead of looking at them all separately. The data analysis section should be written more clearly, and I would suggest reconsidering the analytic plan.

**Authors response:** The reviewer´s comment seems thoughtful. Thus, we have repeated the analyses according to her/his recommendation. Both models led us to the same conclusions:

**A.**

**B.**

**Fig 3. Parallel serial mediation model.** *Cardiorespiratory fitness (independent variable) on academic achievement (dependent variable), through executive functions (mediator variable). Path a, association between independent variable and mediators; path b, association between mediator and dependent variables; path c, total effect between independent and dependent variables; path c´, direct effect of independent variable on dependent variable*.

Finally, we have decided to show separate mediation models for each potential mediator in order to provide the reader a more clearly and simply information. Moreover, we have included also parallel serial mediation (as supplementary material) in order to provide the reader, the complete analyses, although statistical significance disappeared by gender, probably due to the lack of statistical power. Thus, we have included some sentences regarding this concern:

*“Simple mediation models were tested, by introducing independently the three different mediating variables.* *Then, a parallel serial mediation model was tested in which the three domains of executive function (inhibition, cognitive flexibility and working memory) sere simultaneously entered in the model in such a way that each mediator was controlled for the others*” (Page 9, lines 206-210).

*“The mediation model scheme is displayed in Fig 3, and the results of the mediation analysis are shown in Table 3. Inhibition and cognitive flexibility mediated the association between CRF and academic achievement, controlling for age and mother educational level. In the first regression step (path a), CRF was positively related to the executive function domains (p < 0.05). In the second step (path c), the regression coefficients of CRF were positively associated with academic achievement in all mediation models (p < 0.001). In the last regression model (path b), the executive function domains were positively related to the dependent variable (p < 0.001), and the relationship between CRF and academic achievement became weaker (although significant) when the executive function domains were included in the model (path c’). Finally, the indirect effects were statistically significant for inhibition and cognitive flexibility, confirming that executive function domains acted as a partial mediator in these models, except for working memory. The mediation analyses by gender showed that, after controlling for mother educational level, cognitive flexibility was not significant in girls and inhibition in boys. When parallel serial mediation was performed, the results were similar (S2, Fig 4).” (Page 13, line 270-282).*

Minor Issues:

Line 55 – 60: There are many more cognitive processes than the 3 described. Even when considering EF there are many more components to EF than just the 3 that are described.

**Authors response:** Thank you for the comment. The reviewer´s is right. Thus, we have rephrased the sentence as follows:

“*Executive functions are a set of cognitive operations, three of the most studied are inhibition, working memory and cognitive flexibility”* (Page 4, line 58-59).

Line 69: “Indirect effect of executive functions” is not fully accurate. Indirect effect is really the effect of IV through the pathway of mediator

**Authors response:** The reviewer´s is right. We have modified the sentence as follows:

*“These studies have shown a significant positive relationship between physical fitness and academic achievement mediated by executive functions*” (Page 4, line 70-72)

Line 85-89: Discussion of mediation analysis seems unnecessary in the intro and this discussion is somewhat misleading. Mediation cannot determine whether a variable is a true mediator or a confounding variable

**Authors response:** We appreciate this comment, thus, we have removed that paragraph.

Conversely, we have added a brief commentary about the convenience of this analysis in the discussion section:

“*Mediation analysis has been proposed as an statistical procedure suitable for clarifying whether a third variable underlies the relationship between two variables. Thus, this procedure allows us to consider the significant proportion of the influence of the independent variable (CRF) on the dependent variable (academic achievement) takes place through executive functions (mediator) [37]*” (Page 17, line 365-369).

Line 180 – strong effects should read 0.14

**Authors response:** Thank you for the comment. We apologize for the inconvenience. It has been corrected (Page 9, line 193).

208 – “There were no significant differences…” should be rephrased given that there were several differences between gender

**Authors response:** Thank you for the comment. We have rephrased the sentence related to the reviewer’s concern:

“*There were several differences between gender in (…)*” (Page 10, line 216-217)
