# Supplementary material for: Executive functions mediate the relationship between cardiorespiratory fitness and academic achievement in Spanish schoolchildren aged 8 to 11 years
Source: PLoS One. 2020 Apr 10;15(4):e0231246. doi: 10.1371/journal.pone.0231246 (PMC7147757; doi:10.1371/journal.pone.0231246)
Supplement: Attachment — Submitted filename: renamed_3992f.docx [file pone.0231246.s009.docx]

**COMMENTS – Manuscript PONE-D-19-14578R2**

Title: “Executive functions mediate the relationship between cardiorespiratory fitness and academic achievement in Spanish schoolchildren aged 8 to 11 years.”

**Important note: This review pertains only to ‘statistical aspects’ of the study and so ‘****clinical aspects’ [like medical importance, relevance of the study,** **‘clinical significance and implication(s)’ of the whole study, etc.] are to be evaluated [should be assessed] separately/independently.**

Although I recommend to accept the article for publication in this prestigious journal, I request you to note the following [regarding the article quoted in response to my earlier comment saying that “as some authors such as Stillman et al. support the usefulness of these statistical mediation models to test plausibility of causal models, when experimental manipulation (the gold standard for assessing causality) is not available.”]:

Refer to page 6 of

Stillman, C. M., Cohen, J., Lehman, M. E. & Erickson, K. I. Mediators of Physical Activity on Neurocognitive Function: A Review at Multiple Levels of Analysis. Frontiers in Human Neuroscience 10, (2016) which gives the following account:

Cross-sectional studies utilizing statistical mediation provide a theoretical and mechanistic foundation about the relationships between cardiorespiratory fitness, brain, and cognition. However, their correlational nature leaves open the possibility that the observed behavioral and structural fitness-related differences between high and low-fit groups are caused by some unmeasured factor. RCTs are necessary to account for potential selection bias, as well as to establish a direct, causal relationship in humans between aerobic fitness, brain structure, and cognitive functioning.
