# Supplementary material for: Executive functions mediate the relationship between cardiorespiratory fitness and academic achievement in Spanish schoolchildren aged 8 to 11 years
Source: PLoS One. 2020 Apr 10;15(4):e0231246. doi: 10.1371/journal.pone.0231246 (PMC7147757; doi:10.1371/journal.pone.0231246)
Supplement: Attachment — Submitted filename: Response to Reviewers.docx [file pone.0231246.s010.docx]

Dear Academic Editor Joshua Jones,

Please find enclosed a revised version of our manuscript, “Executive functions as a mediator in the relationship between cardiorespiratory fitness and academic achievement in schoolchildren” (PONE-D-19-14578R3). We would like to thank you for giving us the opportunity to revise and improve our manuscript; we also thank the reviewer for the thoughtful and constructive comments. We have valued his or her suggestion in order to improve our research.

Thank you for your attention in this matter.

Mairena Sánchez-López (corresponding author).

**Response to reviewer** **4**:

Although I recommend to accept the article for publication in this prestigious journal, I request you to note the following [regarding the article quoted in response to my earlier comment saying that “as some authors such as Stillman et al. support the usefulness of these statistical mediation models to test plausibility of causal models, when experimental manipulation (the gold standard for assessing causality) is not available.”]:

Refer to page 6 of

Stillman, C. M., Cohen, J., Lehman, M. E. & Erickson, K. I. Mediators of Physical Activity on Neurocognitive Function: A Review at Multiple Levels of Analysis*. Frontiers in Human Neuroscience 10,* (2016) which gives the following account:

Cross-sectional studies utilizing statistical mediation provide a theoretical and mechanistic foundation about the relationships between cardiorespiratory fitness, brain, and cognition. However, their correlational nature leaves open the possibility that the observed behavioral and structural fitness-related differences between high and low-fit groups are caused by some unmeasured factor. RCTs are necessary to account for potential selection bias, as well as to establish a direct, causal relationship in humans between aerobic fitness, brain structure, and cognitive functioning.

**Authors response:** We appreciate the reviewer´s comment. We have considered the difference between what he or she suggested and the idea we were trying to expose. Thus, we have rephrased the following sentences:

*“(…) however, cross-sectional studies utilizing statistical mediation provide a theoretical and mechanistic foundation about the relationships between cardiorespiratory fitness, brain, and cognition. Nevertheless, their correlational nature leaves open the possibility that the observed behavioural and structural fitness-related differences between high and low-fit groups are caused by some unmeasured factor”* (lines 361-365)

*“(…) Randomized controlled trials are necessary to account for potential selection bias, as well as to establish a direct, causal relationship in humans between aerobic fitness, brain structure, and cognitive functioning”* (lines 368-370, page).
